# Visualizing electroluminescence process in light-emitting electrochemical cells

**DOI:** 10.1038/s41467-023-36472-6

**Published:** 2023-03-01

**Authors:** Kosuke Yasuji, Tomo Sakanoue, Fumihiro Yonekawa, Katsuichi Kanemoto

**Affiliations:** 1 Department of Physics, Graduate School of Science, Osaka Metropolitan University, 3-3-138 Sugimoto, Sumiyoshi-ku, Osaka, 558-8585 Japan; 2 Nippon Chemical Industrial Co., Ltd., 9-11-1 Kameido, Koto, Tokyo, 136-8515 Japan; 3 Nambu Yoichiro Institute of Theoretical and Experimental Physics (NITEP), Osaka Metropolitan University, 3-3-138 Sugimoto, Sumiyoshi-ku, Osaka, 558-8585 Japan

**Keywords:** Organic LEDs, Electronic devices

## Abstract

Electroluminescence occurs via recombination reactions between electrons and holes, but these processes have not been directly evaluated. Here, we explore the operation dynamics of ionic liquid-based light-emitting electrochemical cells (LECs) with stable electroluminescence by multi-timescale spectroscopic measurements synchronized with the device operation. Bias-modulation spectroscopy, measuring spectral responses to modulated biases, reveals the bias-dependent behavior of p-doped layers varying from growth to saturation and to recession. The operation dynamics of the LEC is directly visualized by time-resolved bias-modulation spectra, revealing the following findings. Electron injection occurs more slowly than hole injection, causing delay of electroluminescence with respect to the p-doping. N-doping proceeds as the well-grown p-doped layer recedes, which occur while the electroluminescence intensity remains constant. With the growth of n-doped layer, hole injection is reduced due to charge balance, leading to hole-accumulation on the anode, after which LEC operation reaches equilibrium. These spectroscopic techniques are widely applicable to explore the dynamics of electroluminescence-devices.

## Abstract (web summary)

The recombination between electrons and holes in electroluminescence processes has never been directly evaluated. Here, Yasuji et al. directly visualize, with spectroscopic techniques, operation dynamics in light-emitting electrochemical cells during device operation, leading to a rapid device evaluation.

## Introduction

Since the first discovery of prominent electroluminescence (EL) effect^1,2^, EL devices based on organic semiconductors such as organic light-emitting diodes (OLEDs) and light-emitting electrochemical cells (LECs) have attracted much interest and the OLEDs have undergone many technological advancements and even applied to portable displays. An important challenge for further development of EL-based devices is to solve remaining problems to develop stable emitters with high efficiency and long lifetime under normal operating conditions, which requires close examination of the device operating processes. All the EL-based devices operate through essential recombination reactions of electrons and holes to generate luminescent excitons. Unlike simple EL-device structures of the early days, recent EL-based devices usually adopt a multilayer structure with carrier transport and emission layers and/or include host/guest materials or electrolytes for highly efficient device operation^3–6^. In these devices, the exciton generation occurs through multiple processes, making it difficult to scrutinize the operating process. Therefore, the development of measurement methods that can separate multiple processes and track each process is needed, which can lead to further device performance improvements.

The operation of EL-based devices is generally evaluated through the EL-intensity and current, and their ratio gives a measure of the EL-efficiency. However, both quantities are obtained as the final result of multiple processes and cannot provide direct information on the intermediate processes. Since each of the multiple processes usually involves a change in the electronic state, measurement methods enabling electronic state evaluation are desired to examine the device operation processes. Optical spectroscopy in the visible and near infrared (NIR) regions is effective for the electronic state evaluation because it can probe the optical transitions of carriers and excitons generated in organic devices^7–10^. In particular, spectral measurements during the device operation are the most direct, which are often referred to as operando-measurements^11–14^. However, most of the operando-measurements have only been performed as steady-state measurements and they have hardly been applied as time-resolved measurements to study device operation dynamics. There were some reports on the time-resolved measurements of EL spectra^15^ and absorption signals of carriers and excitons induced by the OLED-operation^16–18^. However, time-resolved spectral measurements during device operation have not been established, whereas changes in the electronic state involved by LED operation such as the recombination reactions are observed only by such spectral measurements of the absorption signals of transient species. The time-resolved spectral measurements require high-sensitivity signal detection over a wide wavelength range and thus have been difficult to develop.

As one of the prominent EL-based devices, LECs have recently undergone remarkable progress in device performance^19–24^. Because of their unique features to emit bright EL from devices fabricated with low-cost and scalable solution-based methods typically of a single active layer^25–29^, LECs show promise for the next generation of post-OLED devices. For practical use, however, further improvements of device performance, such as in operational lifetime and efficiency, are required, and the operating mechanism needs to be closely examined. The operating processes and dynamics of LECs have been studied by several experimental methods such as transient analyses of EL intensity and current^30–37^, scanning Kelvin probe microscopy^38–41^, light beam induced current techniques^42–47^, and impedance measurements^48^, some of which enabled direct observation of the growth and spatial shift of doped regions for planar type LECs^40,49–52^. However, it has been shown that the rate of the EL turn-on process in LECs depends almost entirely on the slow ion conduction^28,53–56^, which limits the response rate, thereby masking much faster electronic charge injection during the turn-on process. The slow turn-on process has also been a crucial drawback for the practical application of LEC. Recently, it has been shown that the EL response of LECs becomes much faster when the voltage is raised after waiting under a constant base voltage where ion conduction is almost complete^57^. Raising voltage above the base voltage can also be utilized to study the electronic charge injection process excluding the effect of ion conduction. Thus, the use of the base voltage not only improves the LEC performance but also allows a comprehensive exploration of the LEC operation dynamics over a wide time range from the ion conduction to the electronic charge injection.

In this article, the operating process of LEC is explored by applying spectroscopic measurements combined with the LEC operation on multi-timescales. As a model system of efficient LECs, we employ an LEC composed of a blend by a light-emitting polymer and an ionic liquid (IL)-based electrolyte. It has recently been shown that the LECs with an IL electrolyte of alkylphosphonium phosphate exhibit excellent performance with bright and efficient emission exceeding that of LEDs by the same polymer due to the property of the electrolyte dissolving the polymer^19^. The dissolving property can reduce phase separation between polymers and electrolytes and enhance uniformity inside the LEC, also making it suitable as a simple model system of LEC. In this study, we adopt sandwich-type LECs using the IL electrolyte in contrast to planar type LECs widely used in basic research, because sandwich-type devices could be advantageous for practical applications by utilizing OLED facilities that are already in practical use. Several spectral components with different time scales are truly observed corresponding to the multiple processes expected from the LEC operation such as electrolyte migration, electronic charge injection, and recombination for EL, which are separately examined by controlling a bias on the basis of a constant base voltage. We particularly realize time-resolved spectral measurements to study the electronic charge injections, demonstrating the difference in time evolution between hole injection, electron injection, and EL generation, which enables a comprehensive understanding of LEC-operation. The spectroscopic techniques developed in this study are effective for evaluating device dynamics and are also important as a method that can be widely applied to other EL devices.

## Results and discussion

### Assignment of BM signals

**Fig. 1:**
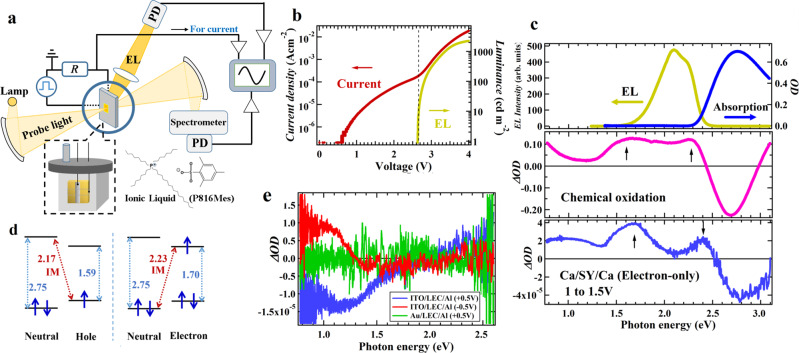
Experimental setup and the fundamental properties of LEC used in this study. **a** Experimental setup employed for the bias-modulation (BM) spectroscopy. A light-emitting electrochemical cell (LEC) consisting of a blend of fluorescent semiconducting polymer, super yellow (SY) poly (para-phenylene vinylene), and ion liquid-based electrolytes (P816Mes) is driven by square wave bias and the change of absorption signal is measured with the electroluminescence (EL) intensity and current. **b** Current density- and luminescence-voltage characteristics of the LEC. The broken line indicates the rise energy point of EL. **c** The steady-state absorption and EL spectra of the LEC (top). The difference absorption spectrum of an SY film after oxidation by iodine gas compared to its stead-state absorption spectrum (middle). BM spectra obtained under the bias from 1.0 to 1.5 V in the Ca/SY/Ca device used as an electron-only diode (bottom). **d** Correlation of the observed transition peaks. IM represents intermolecular optical transitions expected between neutral and charged molecules. **e** BM spectra of the LEC sandwiched by ITO (anode) and Al under 0.5 V and −0.5 V and the LEC sandwiched by Au (anode) and Al under 0.5 V.

A typical experimental setup used in spectral measurements synchronized with the EL operation of LEC is shown in Fig. 1a. An LEC consists of a blend of fluorescent semiconducting polymer, super yellow (SY) poly (para-phenylene vinylene) (PPV), and IL electrolyte, tri-n-octyl-n-hexadecylphosphonium 2,4,6-trimethylbenzenesulfonate (P816Mes, Fig. 1a), which was designed to have good compatibility with SY-PPV to exhibit high performance with stable operation during optical measurements. This LEC is connected to terminals for bias application and current measurements inside an evacuated sample holding container with optical windows for white light illumination from a tungsten lamp or LED. The response of the probe light synchronized with LEC-drive are measured simultaneously with the EL intensity and electrical current. Typical current density- and luminescence-voltage characteristics are shown in Fig. 1b. The current density exhibits two-step rises starting around 0.6 V and 2.7 V, and the EL intensity rises around 2.7 V. The current rise voltages at 0.6 V and 2.7 V correspond to the onset of electrochemical doping and bipolar carrier injection, respectively.

The steady-state absorption spectrum of the SY-LEC has a peak at 2.75 eV and its EL spectrum exhibits appreciable peaks at 2.10 eV and 2.26 eV due to phonon replica by carbon double bonds (Fig. 1c, top). We first present the results of several control experiments conducted to confirm the origin of the spectral signals induced by the operation of SY-LEC which will be shown in the subsequent sections. A pristine SY film without IL electrolytes was oxidized by iodine gas and the difference in its absorption spectrum before and after the oxidation exhibits positive differential optical density (*Δ*OD) peaks at 1.60 eV and 2.28 eV (Fig. 1c, middle). Note that this oxidation was done for a short time to prevent spectral signal contributions from excess iodine gas. A negative *Δ*OD peak also appears at 2.70 eV, close to the absorption transition of SY-LEC, confirming that the oxidation truly occurred. The observed two positive peaks can thus be regarded as the transition peaks of oxidized SY-PPV, i.e., hole carriers.

To know the transition energy of electron carriers, we performed BM experiments from 1.0 to 1.5 V for the Ca/SY/Ca diode without IL electrolytes employed as an electron-only (EO) device with Ca-electrodes for efficient electron injection^41,58^, giving two positive peaks at 1.70 and 2.40 eV (Fig. 1c, bottom). The 1.70 eV-peak is close to the peak of n-type doped SY reported previously^59^ and regarded as the peak of electron carriers in SY. This peak is close to the hole carrier transition at 1.60 eV, which is explained by the reduced energy gap that commonly appears in the polaron transitions of holes and electrons as shown in Fig. 1d. By contrast, the spectral lineshape in the energy region of 2.2 eV to 3.0 eV including the 2.40 eV-peak in the EO device is somewhat similar to that of the first-derivative of steady-state absorption spectrum arising typically from a Stark shift^60^. However, the peaks of the first-derivative spectrum are actually slightly blue-shifted compared to those of the EO device as shown in Fig. S1. Also the negative *Δ*OD peak at 2.79 eV of the EO device is close to the absorption transition of SY, suggesting that the negative *Δ*OD signal arises from photo-bleaching (PB) due to the decrease of neutral components converted to carriers. The peak at 2.40 eV in the EO device is thus concluded as the absorption peaks of electron carriers in SY. These signal assignments to the hole and electron carriers are confirmed by comparison with the BM signals of hole-rich SY diode (ITO/SY/Al) without IL electrolytes in Fig. S2, showing that hole signals appear around 1.55 eV and the fraction of electron signals around 2.2 eV is increased with increasing a bias from 1.5 V to 4.0 V. Also we regard that the transitions around 2.2 to 2.4 eV that appear in the hole and electron carriers are due to intermolecular transitions between a hole or electron carrier and an adjacent neutral molecule (Fig. 1d). In fact, a simple calculation assuming that the central energies of energy gap are the same among the neutral molecule, the hole carriers and electron carriers yields an intermolecular transition energy of 2.17 eV for the hole and 2.23 eV for the electron carrier as shown in Fig. 1d, which are relatively close to the transition energies of hole and electron around 2.2 to 2.4 eV.

In the BM measurements, in addition to the signals of hole and electron carriers, bias-modulation signals are also given by the electrons of the ITO electrode in the ITO (anode)/SY-LEC/Al device. As shown in Fig. 1e, under the bias-modulations of 0 to 0.5 V, negative *Δ*OD signals are observed below 1.7 eV. Since the steady-state absorption spectrum in SY has no absorption transition below 1.7 eV before charge injection, the negative *Δ*OD signals are not the PB signal of SY, suggesting that they are not generated in the SY layer. As control experiments, BM measurements were performed for the Au/SY-LEC/Al device under the same bias conditions, but no significant signal was observed (Fig. 1e). Thus the negative *Δ*OD signal in the ITO/SY-LEC/Al device below 1.7 eV is generated at the ITO electrode, not the Al-electrode. BM experiments were also performed under the reverse bias condition (0 to −0.5 V) for the ITO/SY-LEC/Al device, giving a positive *Δ*OD signal with a lineshape similar to the negative *Δ*OD signal. It was reported that an ITO electrode has a weak Drude absorption band due to free electrons in the near-infrared region typically below 1.5 eV^61,62^. Therefore, when the ITO electrode is positively biased, positive charges on the ITO decreases the number of electrons, resulting in a negative *Δ*OD signal, and when the ITO is negatively biased, the electron signal increases, meaning that the *Δ*OD magnitude below 1.5 eV gives a measure of the amount of charge accumulated on the ITO electrode.

### Slow time regime

The emission process of LEC usually consists of many processes such as ion-migration, doped-layer formation, p-n junction formation, electronic carrier injection and transport, and electron-hole recombination for EL, and the timescales of these individual processes could be different. First, we show the results of spectroscopic measurements during the bias application to the SY-LEC, referred to here as a bias-induced (BI) measurement. The BI measurement probes species generated during the application of a constant bias (see Fig. S3a), enabling us to examine bias-induced electronic state change in the slow time regime. This technique is particularly effective for events associated with ion-migration.

**Fig. 2:**
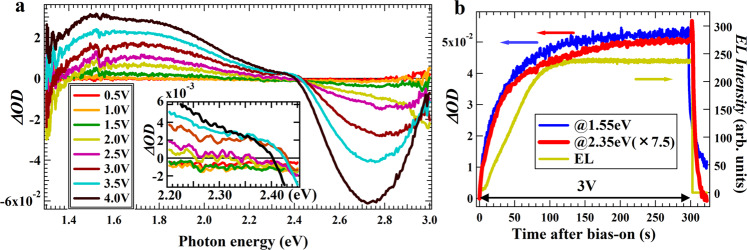
Bias-induced spectroscopic signals in a slow time regime. **a** Bias-induced (BI) spectra obtained for the SY-LEC under steady-state bias application in 0.5 V steps from 0.5 V to 4.0 V. The inset is the enlarged view of spectra from 2.2 to 2.4 eV. These spectra were obtained by recording simultaneously the signals of the entire energy region for 9 s with a CCD detector during the bias application after 180 s of bias application. **b** Comparison of the time-variation among the BI signals at 1.55 eV and 2.35 eV, which are assigned to the signal of hole and electron carriers, respectively, and the EL intensity during the 3V-bias application.

Increasing the constant bias in 0.5 V steps from 0.5 V to 4.0 V under no base bias, the BI signals appear above 1.5 V and their spectra change gradually with the bias-magnitude as shown in Fig. 2a. Since these signals vary in intensity on a timescale of seconds as shown in Fig. 2b, the spectra in Fig. 2a were measured 180 seconds after each bias was applied using a CCD detector capable of simultaneous signal detection from probe light over the wide wavelength range. The positive *Δ*OD signals spreading from 1.3 to 2.4 eV in the spectra are the absorption signals of carriers generated by the bias as discussed in Fig. 1c, while the negative *Δ*OD signals with a peak around 2.7 to 2.8 eV are the PB signals caused by the carrier generation. These slow time scale signals must be caused by charge injection via ion migration, enabling selective detection of carriers generated by IL-induced doping. The carrier signals at all the measured voltages are dominated by two peaks around 1.5 eV and 1.7–1.8 eV, and both peaks appear even below the turn-on voltage of EL (*V*_t_ = 2.7 V) suggesting a monopolar doping regime. In addition to the fact that n-doping is generally more difficult than p-doping in organic semiconductors, the observed spectral structure is similar to that of chemical oxidation in the same region in Fig. 1c. These monopolar doping signals are thus assigned to hole carriers. By contrast, there is no obvious absorption transition ~2.3–2.4 eV below *V*_t_ unlike the result of chemical oxidation. The reason for this is probably that the hole signal is canceled out by other signals including PB, as described later.

We find that the signal around 2.4 eV weakly appears above 3.0 V (Fig. 2a, inset). This weak signal is observed in the bipolar injection region above *V*_t_ and it is unlikely that the hole transitions appear abruptly after *V*_t_. The 2.4 eV signal is thus assigned to the signal from the electron carriers. The reason for this weak signal is probably due to the overlap of other signal components around 2.4 eV. Along with the signal ~2.4 eV, a weak peak appears ~1.7–1.8 eV above 3.0 V, which is close to the peak observed in the EO device in Fig. 1c. There are two types of carrier injections in LECs: dynamic one for the doped layer formation and static one for recombination reactions for EL. The hole signal observed here is mainly given by the p-doped layer formation, whereas in the electron signal, the two injections are difficult to distinguish from the BI measurements alone, and the difference is examined from BM measurements discussed later.

From the results of Fig. 1c and Fig. 2a, the energy positions at 1.55 eV and 2.35 eV can be used as the positions where the bias-dependent features of hole and electron signals can be examined independently, respectively. The growing processes of holes and electrons after the bias is increased from 0 V to 3 V were actually examined at 1.55 eV and 2.35 eV, respectively (Fig. 2b). The time constants of the growing processes were roughly determined to be 40 s and 43 s for the hole and electron signals, respectively (Fig. S4), which are close to each other. These signals observe the process of carrier doping, which consists primarily of charge injection and migration of the electrolytes to the electrodes, and the electrolyte migration is known to be much slower^3,56^, suggesting that the signal growing processes are determined by the electrolyte migration.

Figure 2b also shows the time variation of EL under the same bias condition, and it differs from those of hole and electron signals. The EL signal rises after a delay of about 8 s after the bias-on and exhibits almost constant luminescence after reaching the peak while the hole and electron signals increase gradually. The EL is observed only through the recombination process of holes and electrons, whereas the signals of hole and electron are observed as the total processes consisting of the recombination and the carrier doping including the electrolyte migration. From the difference in the time variation of these signals after the EL reaches the peak, the doping processes in the p- and n-doped layers are found to gradually proceed through the slow electrolyte migration even after the EL reaches an almost steady-state. Furthermore, the observed delay of the EL relative to the carrier signals means that the formation of recombination zone for EL is somewhat delayed relative to the formation of the p-doped and n-doped layers, which is probably because the doped layer formations occur from near the electrodes. This result is obtained for the LEC operation under the bias (3 V) where hole and electron carriers are injected relatively stably, and the dynamic of LEC operation is somewhat different from the case where the LEC is driven under a base bias smaller than 3 V where electron injection is insufficient, as will be discussed later.

### Bias-modulation experiments

**Fig. 3:**
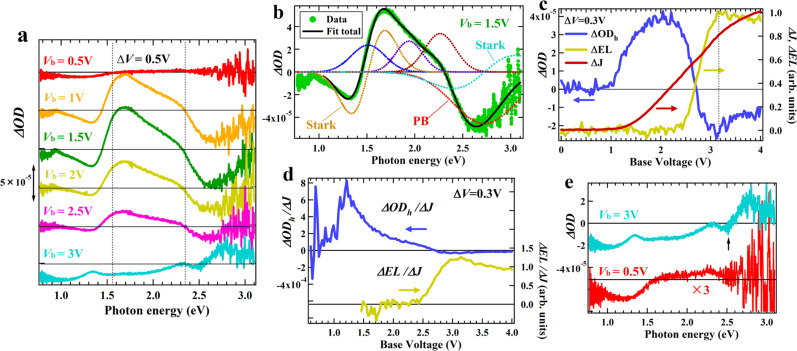
BM spectra obtained under 1 kHz bias-modulation. **a** BM spectra obtained for the SY-LEC at a base voltage (*V*_b_) of 0.5 to 3.0 V under the voltage width (*ΔV*) of 0.5 V. **b** Result of fitting the spectrum at *V*_b_ = 1.5 V with three absorptions and two Stark and one photo-bleaching (PB) components. **c** *V*_b_-dependence of the hole signal intensity at 1.55 eV induced by under the modulation of *ΔV* = 0.3 V (*ΔOD*_*h*_) together with the changes in the current (*ΔJ*) and EL intensity (*ΔEL*) measured simultaneously during the modulation. The sweep rate was 0.01 V/s. **d** *V*_b_-dependence of the hole signal intensity and the change of the EL intensity per the current change during the modulation calculated by *ΔOD*_*h*_/*ΔJ* and *ΔEL*/*ΔJ*, respectively. **e** Comparison of BM spectra of the LEC between under *V*_b_ = 0.5 V and *V*_b_ = 3.0 V.

As shown in the previous section, when a bias is applied from 0 V, multiple processes occur in parallel. In this section, we explore the operation process of LEC based on the results of BM experiments by *ΔV* modulation under a base voltage (*V*_b_) to the LEC using an AC square wave voltage (see Fig. S3b). The frequency of the modulation was 1 kHz, for which, as predicted from the results of the previous section, most of the electrolyte migrations cannot follow. Moreover, the experiment was performed after waiting at least 300 s while applying a constant base bias of *V*_b_. This BM experiment thus mainly observes a change in electronic state due to the carrier injections for chemical doping and EL-recombination reaction that occur after the electrolyte migration is completed at each *V*_b_. The BM spectra obtained for the SY-LEC at *V*_b_ = 0.5 to 3 V in 0.5 V steps under *ΔV* = 0.5 V are shown in Fig. 3a, indicating that the spectra change depending on the magnitude of *V*_b_. Note that the BM measurements provide difference spectra between under *V*_b_ and *V*_b_ + *ΔV*, which is different from the case in the BI measurements that gave spectra under constant bias excitation.

At *V*_b_ = 0.5 V, the spectral response is observed only below 1.5 eV as a negative *Δ*OD signal without PB signals ~2.7 eV. This signal is due to hole accumulation at the ITO electrode as shown in Fig. 1c. Increasing *V*_b_ above 1 V, several noticeable signals appear. A positive peak ~1.55 eV and a negative peak ~2.7 eV are observed, which mainly arise from absorption signals of holes and PB signals, respectively. By contrast, a negative peak appears at 1.35 eV, and its lineshape is different from that of the negative signal of the ITO electrode and has an asymmetric and steep slope unlike normal absorption transitions. This asymmetric lineshape resembles the first-derivative waveform, and we consider that it could originate from the first-derivative signal due to the Stark effect of the hole signal. In fact, it has been reported that in devices containing a large number of ions or carriers, the electric field from these charges causes a Stark signal^63,64^. In the present case, the injected holes could be subjected to an electric field from the anions of IL, causing the Stark shift of the absorption transition together with the generation of the absorption signals.

Figure 3b depicts the result of fitting the spectrum at *V*_b_ = 1.5 V with the hole absorption transition at 1.51 eV and the PB signal at 2.73 eV, each of which consists of the sum of the Gaussian absorption lineshape and its first-derivative with the same peak position and line width, as well as the Gaussian absorption lineshape of hole signals with peaks at 1.92 eV and 2.26 eV. The 1.5 V-spectrum is well reproduced, suggesting that the Stark signal is partly included in the hole and PB transitions, and that a dynamic transition shift occurs in the hole absorption together with the hole injection. Also, although there was no apparent hole signal around 2.3 eV in the BI measurement spectra in Fig. 2a, this spectral fit suggests the presence of hole signal around the energy. This hole signal is mostly canceled by the PB signal and the Stark signal of the ground-state absorption but may be responsible for the weak shoulder peak at 2.35 eV in Fig. 3a.

There is no appreciable change in the lineshape of BM spectra under from *V*_b_ = 1 to 2.5 V, but the spectrum intensity decreases after *V*_b_ = 1.5 V, while the current in the current-voltage characteristics of Fig. 1b increases with *V*_b_ in the voltage region. Moreover, at *V*_b_ = 3 V where the EL is pronounced, the hole signal around 1.55 eV and PB signal around 2.75 eV are inverted. The observed *V*_b_-dependent features are discussed based on the *V*_b_-dependence of the hole signal intensity, which was measured at 1.55 eV by sweeping *V*_b_ under the constant modulation of *ΔV* = 0.3 V (see Fig. S3b) simultaneously with the current and EL intensity generated for the same *ΔV*–modulation (Fig. 3c). Note that the current was measured as a steady-state value during the square wave modulation using a boxcar integrator in order to exclude the contribution from a displacement current (Fig. S5) that is given by charging carrier components preceding the injection. The current change (*ΔJ*) and the BM signal of hole (*ΔOD*_*h*_) rise at *V*_b_ close to each other ~1.1 V, but, while *ΔJ* increases monotonously with increasing *V*_b_, *ΔOD*_*h*_ peaks around *V*_b_ = 2.1 V and then decreases rapidly. After *V*_b_ = 2.7 V, *ΔOD*_*h*_ decreases even to negative values, being consistent with the spectrum in Fig. 3a. We consider that hole carriers with a short residence time in the active layer, such as those that immediately pass through the p-doped layer toward the cathode or recombine for EL, do not contribute sufficiently to the signal intensity of *ΔOD*_*h*_. Thus, the *ΔOD*_*h*_-intensity is mainly determined by the holes forming the p-doped layer, indicating that the growth of the p-doped layer practically stops at *V*_b_ = 2.6 V where *ΔOD*_*h*_ = 0.

Figure 3d shows the *ΔOD*_*h*_/*ΔJ* and *ΔEL*/*ΔJ* calculated from the results of Fig. 3c. *ΔOD*_*h*_/*ΔJ* corresponds to the relative ratio of hole carriers used for the p-doped layer formation per the injected charge. After the initial rise from around 0.5 V, *ΔOD*_*h*_/*ΔJ* exhibits a peak immediately at 1.2 V and then decreases gently. In particular, this decrease occurs before the onset of *ΔEL*/*ΔJ*, suggesting that it is not due to an increase in the fraction of holes consumed for recombination, but to an increase in the fraction of holes that pass through the p-doped layer without being consumed in the formation of the p-doped layer. We thus draw the following picture of the hole doping process. At the *ΔOD*_*h*_/*ΔJ* peak of 1.2 V, an effective hole-transport path to the cathode is formed. Increasing *V*_b_, the transport path grows gradually, which further decreases *ΔOD*_*h*_/*ΔJ*, and the doping process is mostly completed at *V*_b_ = 2.6 V where *ΔOD*_*h*_ = 0.

*ΔEL* in Fig. 3c starts to increase at *V*_b_ = 2.4 V (note that the actual rise voltage in this measurement is *V*_b_ + 0.3 V and coincides with *V*_t_ = 2.7 V for *V*_b_ = 2.4 V), from which, by contrast, *ΔOD*_*h*_ starts to decrease. Also the voltage of positive *ΔEL* peak (*V*_b_ = 3.15 V) in Fig. 3c is close to that of the negative peak of *ΔOD*_*h*_ (3.10 V), suggesting that the decrease of *ΔOD*_*h*_ is related to the increase of *ΔEL. ΔEL*/*ΔJ* in Fig. 3d provides information on the relative EL efficiency in the *ΔV*-modulation range, and we emphasize that *ΔEL*/*ΔJ* has a peak around *V*_b_ = 3.0–3.2 V, where *ΔOD*_*h*_/*ΔJ* is very small. This result reveals the characteristics of EL-efficiency in LECs. Namely, since the intensity of *ΔOD*_*h*_ is mainly determined by the density of holes forming the p-doped layer, the injected holes are not consumed in the p-doped layer formation around the peak voltage of *ΔEL*/*ΔJ* but are effectively consumed in the EL-recombination, resulting in the peak of EL efficiency. Also, in the region where *ΔOD*_*h*_ < 0 (*V*_b_ > 2.7 V), the consumption of holes exceeds the injection. The appearance of this region should be due to a temporary decrease of the hole density in the p-doped layer due to the formation of a depletion layer, caused by the growth of p-n junctions as the electron doping progresses. Thus, the present spectral measurement is also useful to explore the formation process of the depletion layer. In fact, the depletion layer formation could be completed when *ΔOD*_*h*_ reaches its minimum at *V*_b_ = 3.1 V, and above the voltage, *ΔOD*_*h*_ increases again toward the balance between the injection and consumption of hole.

Based on the results of the above experiments, the origin of the spectrum observed at *V*_b_ = 3.0 V in Fig. 3a is discussed again. In addition to the inverted transitions of the hole absorption and the PB signal, this spectrum exhibits several transition peaks. First, it contains a peak at 2.5 eV, which is close to that of the ground-sate (GS) Stark signal in Fig. 3b. Such a Stark component should thus be included actually in the BM spectra, and this Stark signal is not inverted unlike the hole transitions, which corresponds well with the expected feature of GS Stark signal that arises from an electric field by the applied bias and is not related to the hole transition. Additionally, in the 3V-spectrum, a negative *ΔOD* signal is pronounced ~1.0 eV and its lineshape is similar to that of the 0.5V-spectrum as compared in Fig. 3e. This similarity suggests that the some of hole carriers may not be injected into the active layer at 3 V but accumulated on the ITO electrode under a bias above *V*_t_, which is discussed later. The 3V-spectrum in Fig. 3e also exhibits a positive peak at 2.34 eV. The spectra from *V*_b_ = 1 V to 2.5 V n Fig. 3a also have a weak shoulder peak at a similar energy position, but the peak of the 3V-spectrum was not inverted unlike hole transitions but is rather pronounced at the voltage. Therefore, the peak at 2.34 eV is not directly related to the hole carriers but is attributed to the electron carriers, as suggested by the BI spectra above 3 V in Fig. 2a.

It has been reported that the relative EL efficiency of LECs calculated by dividing the EL intensity by the current typically has a roll-off after reaching its peak^56^, and the roll-off has been explained as being caused by reabsorption due to carrier transitions^65,66^ or exciton quenching due to recombination between carriers and excitons^37,57^. In both mechanisms, the energy overlap between the emission and carrier transitions is a key factor for the roll-off to occur. An efficiency roll-off is found in *ΔEL*/*ΔJ* above 3.2 V in Fig. 3d. The EL of the SY-LEC is mainly observed in the energy region of 1.9–2.4 eV, where the hole absorption is unlikely to cause the roll-off as it is rather reduced in the 3V-spectrum. By contrast, the transition of electrons at 2.35 eV has an energy overlap with the EL, and its intensity increases in the 3V-spectrum. Moreover, the EL was measured from the ITO side, and the EL output includes the light generated in the depletion layer that passes through the n-doped layer twice via reflection at the Al-electrode. The electron transition can thus be one of the causes of the observed roll-off.

### Dynamics of LED operation

**Fig. 4:**
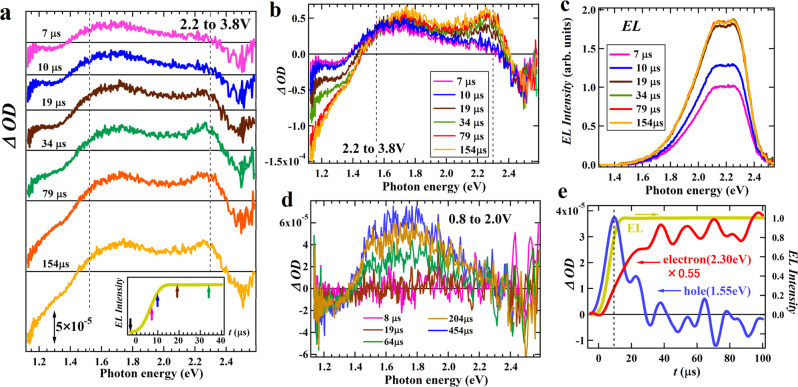
Operation dynamics of LEC. **a** Time-resolved BM spectra of the SY-LEC obtained when the bias is raised from 2.2 V to 3.8 V where the baselines were shifted for each spectrum represented by the horizontal straight line. The inset is the time evolution of EL intensity, with arrows indicating the baseline point (black) and several time points at which the BM spectrum was measured (7 μs, 10 μs, 19 μs, and 34 μs). **b** The same time-resolved BM spectra as in **a** represented with a unified baseline. **c** Time-resolved EL spectra measured at the same times as in the time-resolved absorption spectra in **a** and **b**. **d** Time-resolved BM spectra of the SY-LEC measured when the bias is increased from 0.8 V to 2.0 V. **e** Transient responses of the BM signals at 1.55 eV and 2.30 eV assigned to hole and electron carriers, respectively, and EL.

As shown in the previous sections, the EL operation in the LEC consists of many processes such as the injection and doping of holes and electrons, the carrier and electrolyte transports, and the recombination for emission, and they may occur in parallel in the time-domain. In this section, we examine the dynamics of LEC operation considering the correlation among these processes directly through time-resolved (TR) BM measurements. Unlike typical pump-probe experiments using photoexcitation, where the intensity of photoexcitation can be increased for small signal intensities, the *ΔOD* values in the BM measurements are as small as 10^−4^ to 10^−5^ under actual operational bias as shown in the previous section, and the TR-BM measurements require a sensitive measurement system enabling measurements of the time variation of small signals. We constructed a measurement system consisting of a high-resolution oscilloscope and a low-pass filter that enables detection of transient BM signals with high-sensitivity (see Experiments section for details). Figures 4a and 4b show the actual results of TR spectral measurements for the bias raised from 2.2 V to 3.8 V (see Fig. S3c): the former and latter panels are represented with the baselines of each spectrum shifted and with the same baseline, respectively. Note that the electrolyte transport is nearly completed at 2.2 V. The inset of Fig. 4a shows the EL intensity at the time when each TR-BM spectrum was measured, and the TR-EL spectrum at each time is shown in Fig. 4c. The lineshape of the EL spectra is found to be unchanged (Fig. S6), indicating that the same emissive excitons are generated within the time range.

The absorption signals of hole carriers appear around 1.5 to 1.7 eV at the 7 μs-spectrum but their signal intensities hardly change after 7*μ*s. By contrast, for the bias raised from 0.8 V to 2.0 V where no EL occurs, the hole signal intensity increases with time without significant lineshape change as shown in Fig. 4d. Thus, the behavior of hole signal intensity in Fig. 4a, b that hardly changes with time is a feature found in the bias region above *V*_t_. Also, despite the hole intensity reaching nearly steady-state at 7 μs, the EL intensity at 7 μs is about 55% of the steady-state intensity which is reached steady-state around 13 μs, meaning that the EL occurs with a slight delay to the hole signal. Figure 4e compares directly the time responses between the hole signal measured at 1.55 eV and the EL, demonstrating that the hole signal rises earlier. Note that the hole signal in Fig. 4e decreases immediately after the initial increase, which was not observed in Fig. 4a, b. However, we also obtained TR-BM spectra with the hole signal decreased quickly after the initial rise, corresponding to the case in Fig. 4e, from a different SY-LEC (Fig. S7). Thus, the difference in the hole signal dynamics probably arises from a fluctuation in the amount of IL electrolytes within the p-doped layer due to the gradual migration of IL electrolytes, which proceeds slowly on a timescale of minutes during repeated measurements. Actually, in the case of hole signals decreased after the initial rise, the hole signal increased gradually after the decrease (Fig. S8), suggesting that slow electrolyte migration for p-doping follows the hole decrease. In both cases of hole signals, it is common that the hole signal precedes the EL.

The TR spectra in Fig. 4a, b also exhibit a pronounced peak from electrons around 2.28 eV, which also accompanies a broad peak at 1.73 eV, being consistent with the spectrum of EO device. We note that the peak position of the electron signal at 2.28 eV is close to one EL peak, but this electron signal is not given by the EL signal because the electron peak in Fig. S7 is observed at 2.35 eV, differing from the EL peak position, and the other EL peak at 2.10 eV is not observed in the TR spectra in Fig. 4a, b. Unlike the characteristics of hole transitions, the electron signal appears from the 19μs-spectrum and its intensity increases even after the EL reaches steady state. The TR spectra also exhibit a negative peak ~2.5 eV, which hardly changes with time. This feature is typical for the Stark transition originating from the applied electric field, being consistent with the assignment described earlier. The transition ~1.2 to 1.3 eV due to the holes accumulated on the ITO electrode also appears appreciably at the 19 μs spectrum, and is found to grow significantly with time. Thus, the assignments of all observed signals in the TR spectra are clear, allowing the LEC operation to be directly tracked in the time domain.

**Fig. 5:**
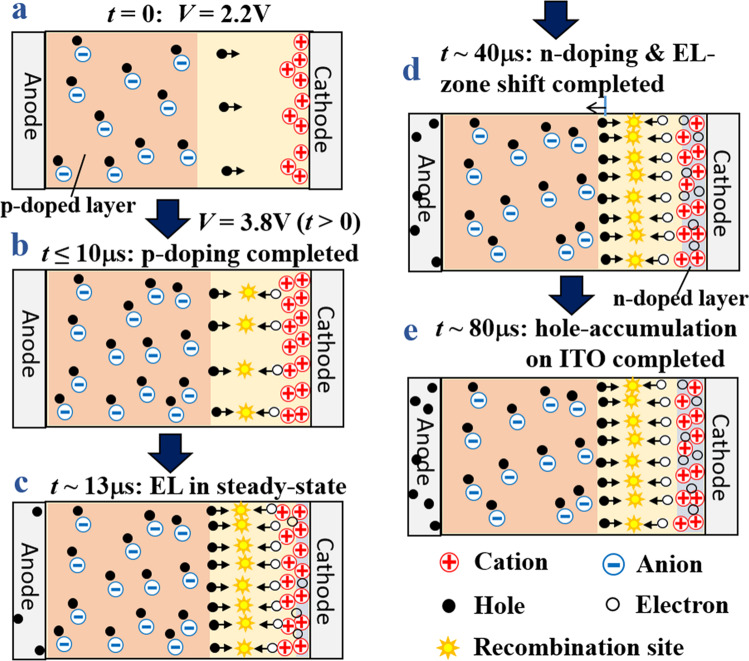
Schematic of operation dynamics of LEC. Taking the experimental situation in Fig. 4 as an example, **a** shows the standby state under a bias at 2.2 V, and the bias is raised to 3.8 V in **b**, which is then followed by **c**–**e** with the passage of time. Note that some ions in the non-doped region are not depicted in the figures for simplicity.

Based on the results of these TR measurements, the dynamics of the LEC operation is discussed in Fig. 5. Under the bias increased from the base bias of 2.2 V to 3.8 V, both p-doing injection and LED-type injection occur, and both may contribute to the increase in the hole signal intensity. Since the LED-type injection should occur after sufficient p-doing layer formation, the immediate increase of hole signal after bias application is due to the p-doing layer formation. After the formation, the hole signal intensity in Fig. 4a, b changes hardly with time despite the increase in the EL intensity, indicating that the hole density is balanced by the LED-type injection and consumption by recombination. This balance suggests an ideal LED-type operation in which most of the injected holes are consumed by the recombination after the initial p-doping layer formation. By contrast, in the case of the hole signal decreasing quickly after the initial rise in Fig. 4e, the decrease of hole signal is not due to the recombination because, during the hole intensity decrease, hole injection occurs continuously and the EL intensity is nearly constant. Related to the decrease in the hole signal in Fig. 3e, such a dynamic decrease of hole intensity should be due to the reduction in the p-doped layer caused by the depletion layer formation, suggesting that the retreat of the p-doped layer is completed on a time scale of 20-30μs.

For the result that the hole signal precedes EL, one might consider the following two reasons. One reason is that electron injection could be slower than hole injection, and the slower electron injection delays the recombination for EL. The other reason is that the electron injection occurs at a similar rate to the hole injection, and the diffusion of injected holes to the recombination zone through the p-doped layer could cause a time delay between the hole injection and the EL recombination. However, in the latter case, the electron carriers waiting for the injected holes to reach the recombination zone must be observed before the EL. In fact, Fig. 4e demonstrates that the intensity of the electron signal at 2.30 eV is small at the initial stage of raising the bias. Therefore, the result of the hole signal preceding EL indicates that the electron injection is slower than the hole injection, meaning that the response rate of EL is nearly determined by the rate of electron injection in this LEC.

The result of the small intensity of electron signal at the initial stage of bias application in Fig. 4e also indicates that most of the injected electrons are initially consumed in the EL-recombination without forming the n-doped layer. Thus, at 2.2 V, the n-doped layer is very thin and the thicker p-doped layer could reach near the interface with the cathode (Fig. 5a), which enables immediate capture of the injected electrons at 3.8 V (Fig. 5b, c). The subsequent gradual increase in the electron signal corresponds to the growth of the n-doped layer, which is almost completed in about 40*μ*s as suggested from Fig. 4e (Fig. 5d). The n-doped layer growth involves a gradual shift of the recombination zone to the p-doped layer side on a time scale where the electron signal increases: in fact, the increase rate of the electron signal and the decrease rate of the hole signal in Fig. 4e are similar. Such a zone shift is enhanced by increasing the bias, leading to the observed decrease of hole signal by increasing *V*_b_ in Fig. 3c. This operation dynamics is different from the case of LEC operation starting from zero bias, where the EL is somewhat delayed relative to the formations of p- and n-doped layers as shown in Fig. 2b. This difference arises probably because in the zero bias case, n-doping proceeds from near the cathode by slow IL electrolyte migration without consumption by recombination, whereas in the operation after the base bias of 2.2 V, the injected electron carriers are immediately captured by hole carriers in the p-doped layer spreading near the cathode.

The operation processes of LEC have often been described by the electrochemical (EC) model^3,67^ and the electrodynamic (ED) model^68–70^, which assume the LEC operation under the sufficient formations of doped layers consisting of electronic carriers and electrolytes and electric double layers (EDLs), respectively. It is known that the applicability of the two models depends on the degree of ohmic contact and that they can coexist under an actual LEC operation^40,41^. In fact, the observed spectral signals are primarily given by stable carriers, which probably arise from doped layers consisting of electronic carriers and electrolytes, suggesting the suitability of the EC model, whereas the delayed EL response relative to the doped layer formation when starting from zero bias suggests the formation of EDLs as well as the doped layers, suggesting the suitability of the ED model. Thus, in the operation starting from zero bias, the cases of the two models coexist. moreover, in the operation starting from the base bias, LECs operate after the sufficient formations of the doped layers and EDLs, also suggesting the coexistence of the two model cases.

We here pay attention to the behavior of hole carriers during the growth of n-doped layer. The hole signal was constant or decreased during the n-layer growth, meaning that there is no accumulation of hole carriers inside the LEC during the process. We consider that the charge of hole carriers corresponding to that of the electron carriers increased with the n-layer growth accumulates on the ITO electrode (Fig. 5d), which corresponds to the observation of hole signals from the ITO electrode. Actually, in Fig. 4a and b, the signal of the ITO around 1.2 eV exhibits a time variation similar to the electron signal. However, we note that the signals of ITO saturate at about 80 *μ*s (Fig. 4b), which is longer than the saturation time of electron signals. The difference in the saturation time between these signals occurs probably to regulate the charge balance after the formation of the n-doped layer, which finally reaches charge equilibrium at 80 *μ*s (Fig. 5e).

These are the operation dynamics of the LECs as revealed by the TR-BM spectroscopy. In this study, all the measurements were performed under unbalanced bipolar charge injection under the base bias where the growth of the n-doped layer is insufficient, while this LEC operation is important to the present LEC for fast response and reduced device degradation. In the case of sufficient n-doped layer growth, which will be achieved under a larger base bias, the dynamics of LEC operation could be different and should be comprehensively discussed considering the contribution of temperature rise and possible degradation, which is a future step.

We developed an evaluation of the LEC operation dynamics using BM spectroscopy. Conventional evaluation methods for light emitting devices are almost limited to current and EL intensity measurements, but the methods in this study enabled direct spectroscopic evaluation of recombination processes through hole and electron signals. In particular, current-based measurements, including impedance measurements, cannot separate the contributions of holes and electrons, but the BM method was able to separate them by clarifying the difference in the injection rate of holes and electrons, thereby revealing the operation dynamics leading to steady EL generation. The TR-BM method also showed the recession of the p-doped layer occurring on a fast timescale, and the recession was shown to proceed with increasing bias from BM spectroscopy by lock-in techniques. These results correspond to a direct demonstration that the recession of p-doped layer occurs, which has been suggested previously but not directly demonstrated. These spectroscopic techniques can thus provide new information that cannot be extracted by other experiments and are also applicable to other light-emitting devices including OLEDs. We expect that these techniques will be utilized in the future for rapid evaluation systems enabling direct identification of processes that degrade the performance of light-emitting devices.

## Methods

### Device fabrication

The toluene solutions of SY-PPV (6 mg mL^−1^) and IL-based electrolyte (P816Mes, Nippon Chemical Industrial Co., Ltd.: 9 mg mL^−1^) were prepared and mixed with a weight ratio of 6:1, corresponding to 4:1 in the weight ratio of SY-PPV: P816Mes. The blend solution was spin-coated on the ITO electrode (anode) at 1500 rpm for 30 s and annealed at 150 °C for 30 min. The thickness of the blend film was about 100 nm. An Al layer was then deposited on the blend film by vacuum evaporation at a thickness of 20 nm to form a semitransparent cathode. The active area of the device was 7 × 7 mm^2^. Only the LECs exceeding a luminance of 300 cd/m^2^ under a bias of 3 V, corresponding to ~2.3 × 10^3 ^cd/m^2^ at 4 V, were used for optical measurements. All of the above procedures and introduction into the sample holding container for optical measurements were done in a nitrogen-filled glove box. This treatment of devices allows for stable bias operation during optical measurements.

### Optical measurements

The bias-induced spectra during the bias application were measured by recording the intensity of the probe light transmitted through the LEC using a CCD detector after waiting more than 180 s of bias application. The probe light was produced from a white LED. The BM measurements were performed by applying a square-wave AC voltage to the LECs and detecting the modulation signals of the transmitted probe light from the tungsten lamp synchronized with the frequency of the AC voltage (1 kHz) using a lock-in technique. The modulation bias (±1/2 *ΔV*) was applied after waiting at least 300 s while applying a constant bias value of *V*_b_ + 1/2 *ΔV*, which corresponds to experiments to examine the response to the bias modulation of *ΔV* under the base bias *V*_b_. The probe light was detected by a Si photodiode for the visible region and an InGaAs detector for the NIR region and then amplified by a preamplifier (Femto, DHPCA-100). The *V*_b_-dependence of the BM signal was measured at 1.55 eV for the fixed *ΔV* (0.3 V) simultaneously with the EL-intensity and current. A colored glass filter and an interference filter were then used to block the visible light of the probe light from the lamp and to selectively detect the EL, respectively. In the current measurements, a saturated current was selectively. The current induced by applying *ΔV* was measured by picking up a saturated current in the transient current using a gate function of a boxcar integrator. The total time for sweeping from *V*_b_ = 0 to 4 V was 400 s (0.01 V/s), which is expected to be sufficient time for the effect of electrolyte ion migration to reach a steady-state.

The TR-BM spectra were measured by combining wavelength sweeps and high-resolution signal detection using a digital oscilloscope (Teledyne Lecroy, HD 4096) for the probe light synchronized with the applied square AC voltage with a repetition frequency of 1 kHz. The signal from the probe light was detected by the photodiodes described above and amplified by the high-speed preamplifier described above. We note that, in order to obtain the spectra, stable device operation with negligible impact of degradation during long-time data acquisition are required. The rise time of the time-resolved absorption signals was found to be delayed due to the amplification by about 2 to 3 microseconds. Although the rise rate of fast signal was not accurately determined by this measurement, the absorption signals and EL at each wavelength were measured under the same amplification conditions, meaning that the relative relationship between the absorption signals and EL could be recorded correctly. The time-resolved spectra at each time after the bias was applied (*t*) were obtained by subtracting the transmitted light intensity at *t* = −0.5 μs from the transmitted light signal intensity at each wavelength. All measurements were performed at room temperature under vacuum conditions.

## Supplementary information

Supplementary Information

## Data Availability

The data that support the findings of this study are available from the corresponding author upon request.
