# Supplementary material for: Visualizing electroluminescence process in light-emitting electrochemical cells
Source: Nat Commun. 2023 Mar 1;14:992. doi: 10.1038/s41467-023-36472-6 (PMC9977921; doi:10.1038/s41467-023-36472-6)
Supplement: Supplementary file 1 — Supplementary Information [file 41467_2023_36472_MOESM1_ESM.pdf]

Supplementary Information for

Visualizing electroluminescence process in light-emitting electrochemical cells.

Kosuke Yasuji[1], Tomo Sakanoue[2], Fumihiro Yonekawa[2], Katsuichi Kanemoto[1,3]*

1 Department of Physics, Graduate School of Science, Osaka Metropolitan University
3-3-138 Sugimoto, Sumiyoshi-ku, Osaka 558-8585, Japan.

2 *Nippon Chemical Industrial Co., Ltd., 9-11-1 Kameido, Koto, Tokyo 136-8515, Japan.*

3 *Nambu Yoichiro Institute of Theoretical and Experimental Physics (NITEP), Osaka Metropolitan University, 3-3-138 Sugimoto, Sumiyoshi-ku, Osaka 558-8585, Japan*

This file includes below:

1. Comparison of BM spectrum of electron-only diode and the first-derivative spectrum.
2. BM spectra of SY diode
3. Bias used for each spectroscopy
4. Fit results for the signal increase process.
5. Explanation of the current measurement in the modulation experiments.
6. Comparison of time-resolved EL spectra.
7. Time-resolved BM spectra.
8. Enlarged view of the transient response of hole signal.

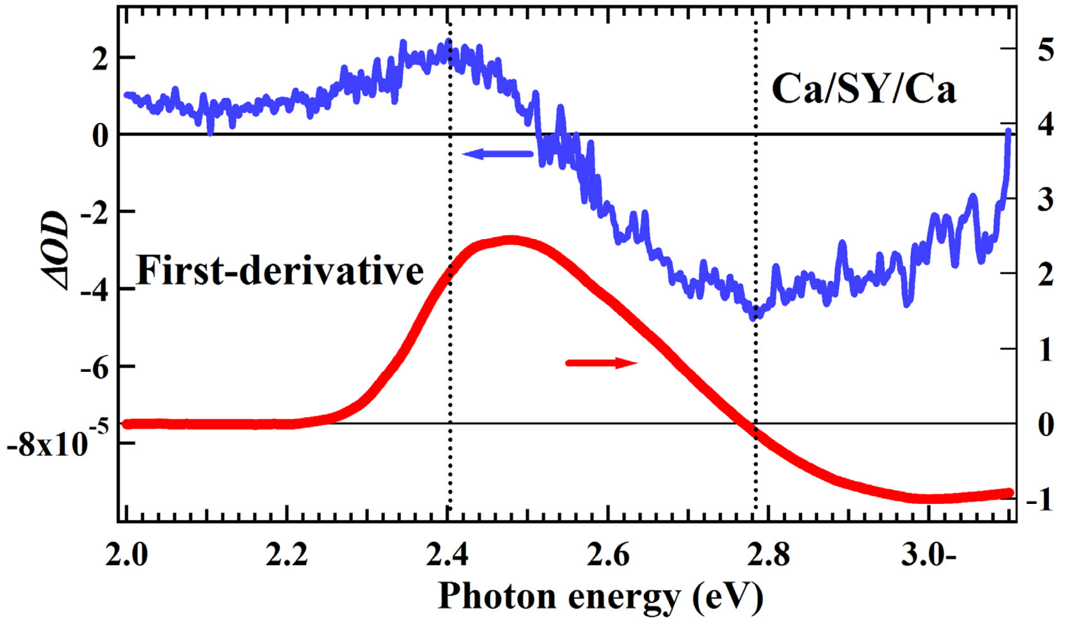

Figure S1 | Comparison of BM spectrum of electron-only diode and the first-derivative spectrum. This figure compares the bias-modulation (BM) spectrum of the electron-only device (Ca/Super yellow (SY)/Ca) with the first-derivative of the ground-state absorption spectrum of SY.

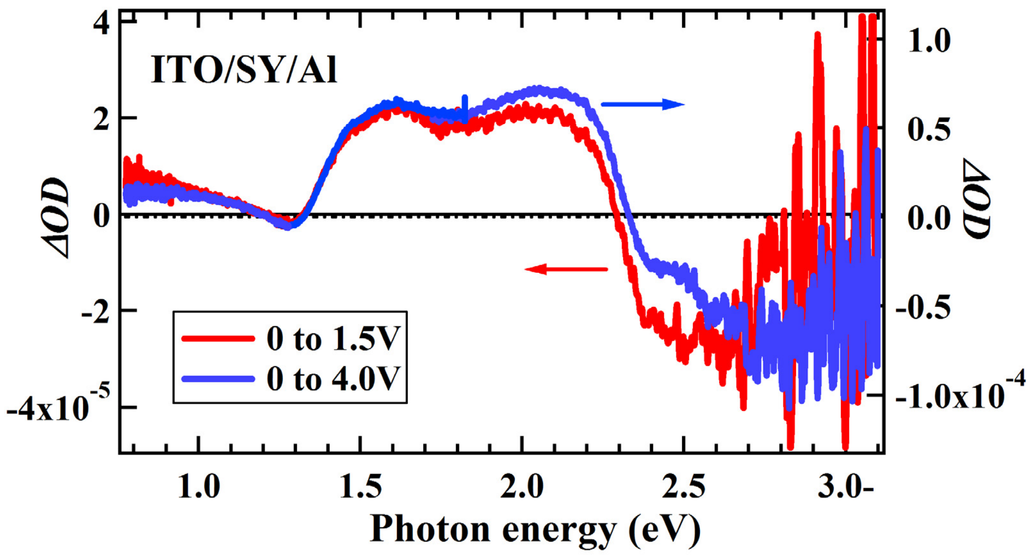

Figure S2 | BM spectra of SY diode. This panel compares the spectra of BM measurements for the hole-rich SY diode of ITO/SY/Al without ion-liquid electrolytes between ΔV= 1.5V and 4.0V under the base bias of 0V.

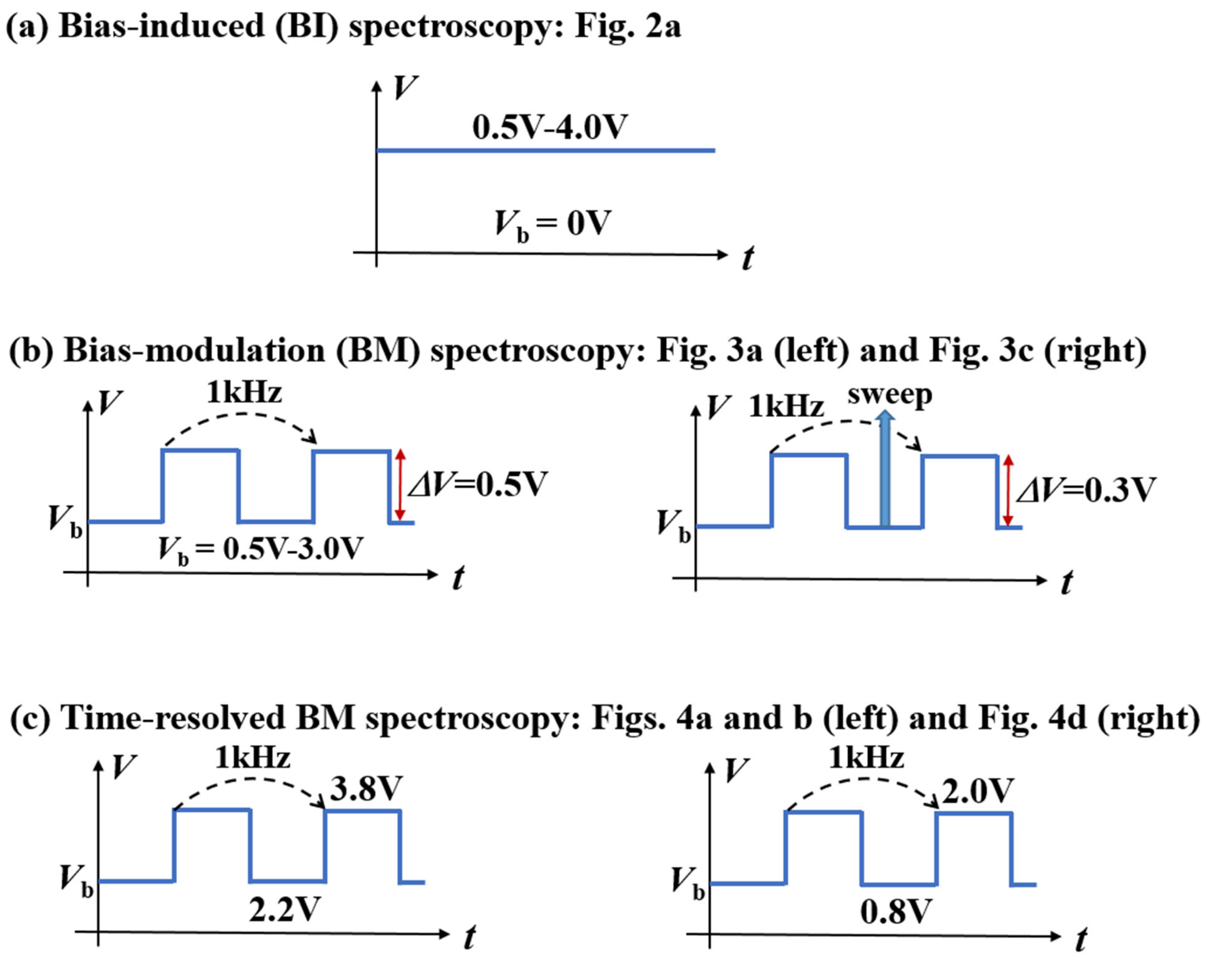

Figure S3 | Bias used for each spectroscopy: a Constant bias used for bias-induced (BI) measurements. **b** Square-wave AC bias (1kHz) used for bias-modulation (BM) measurements. **c** Square-wave AC bias (1kHz) used for time-resolved BM measurements.

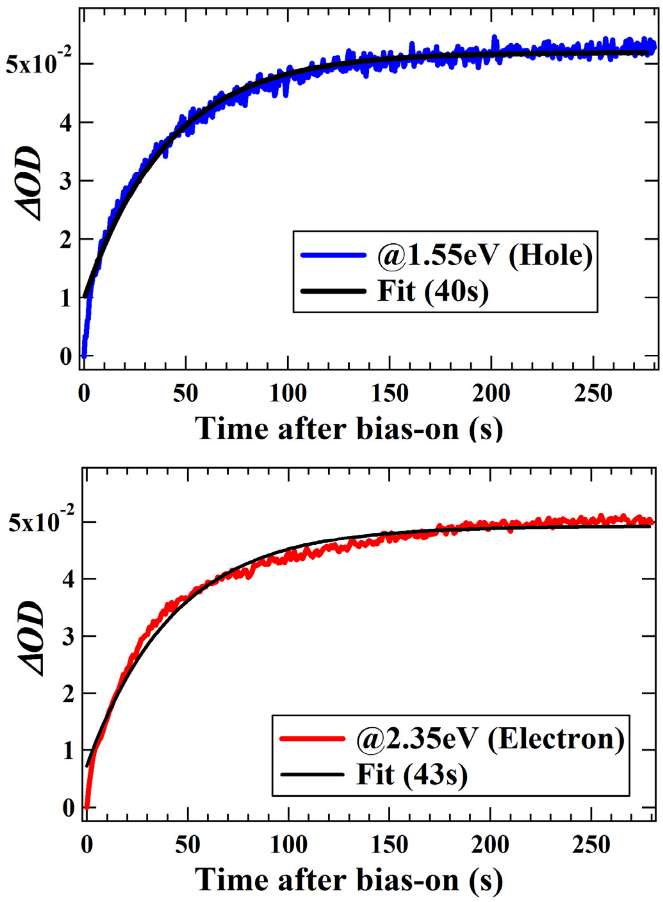

Figure S4 | Fit results for the signal increase process. The results of the signal increasing process during the 3V-bias application shown in Fig. 2b were fitted with a single exponential curve. The best-fit results for the bias-induced signals at 1.55eV and 2.35eV were 40s and 43s respectively. Note that, since the signal increasing processes are mainly determined by the migration of ionic liquid-electrolytes, which may not yield an exponential time response, the obtained time constants are used as an indication of time response rate.

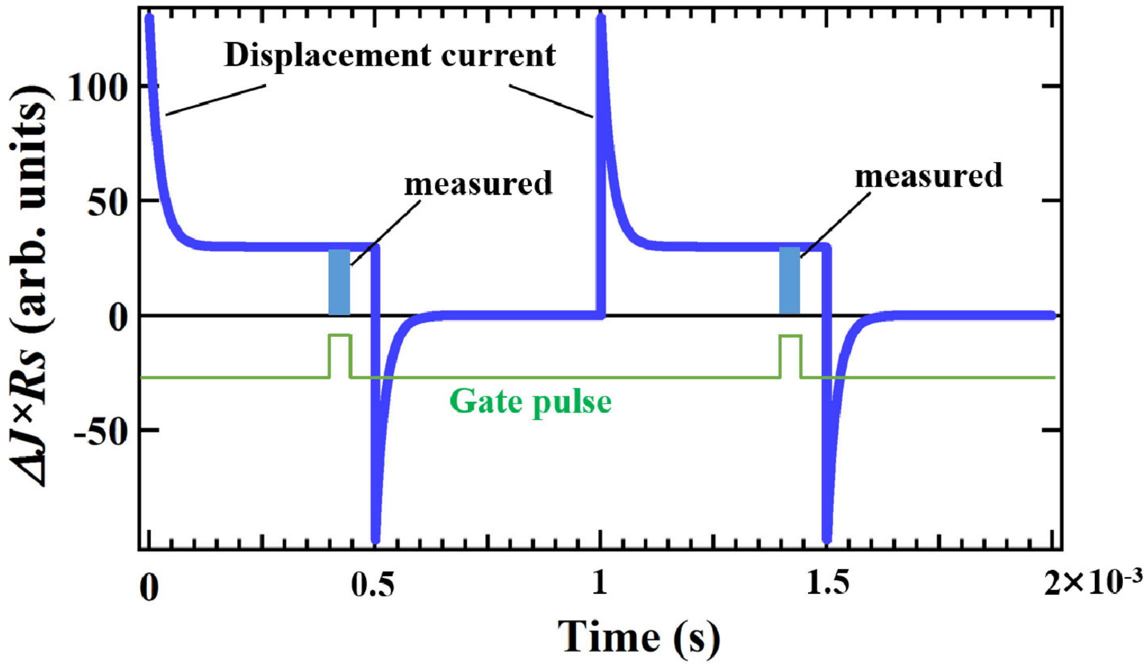

Figure S5 | Explanation of the current measurement in the modulation experiments. Applying a squared AC bias to an LEC, an obtained current including a displacement current that occurs at the moments of bias-on and off and decays depending on a circuit constant. In order to remove the contribution of the displacement current, the current under the application of squared bias was measured after reaching a steady-state value where the displacement current is negligibly small, using the gate function of the boxcar integrator.

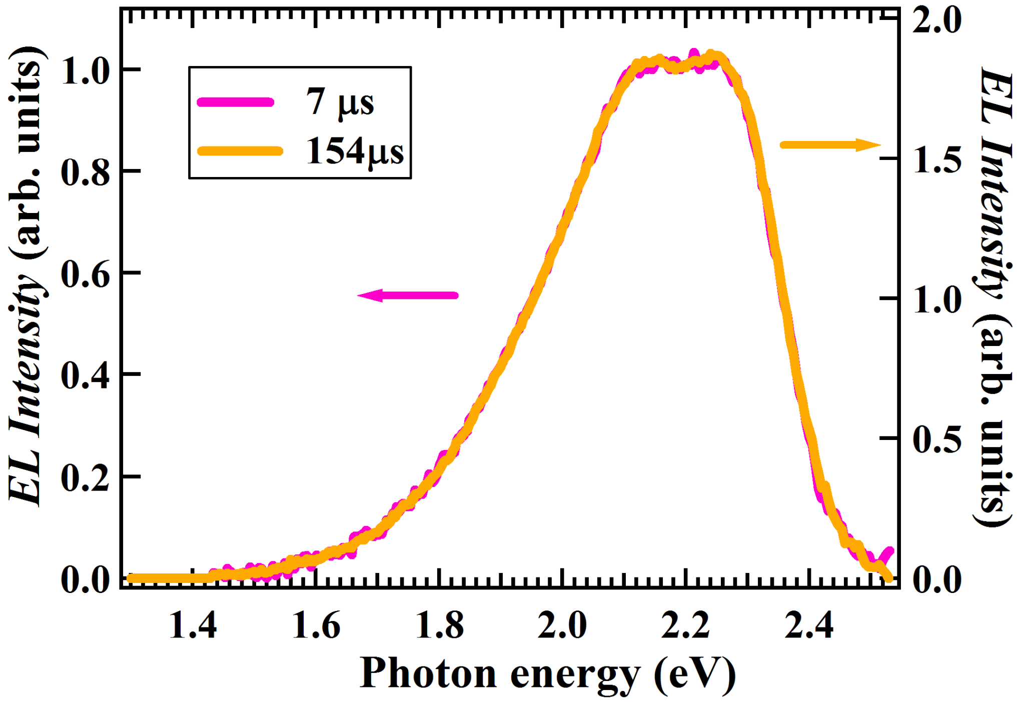

Figure S6 | Comparison of time-resolved EL spectra. This figure compares the time-resolved EL spectra in Figure 4c obtained at 7 and 154 μs after the increase of bias from 2.2 V to 3.8 V and indicates the two spectra coincide with each other.

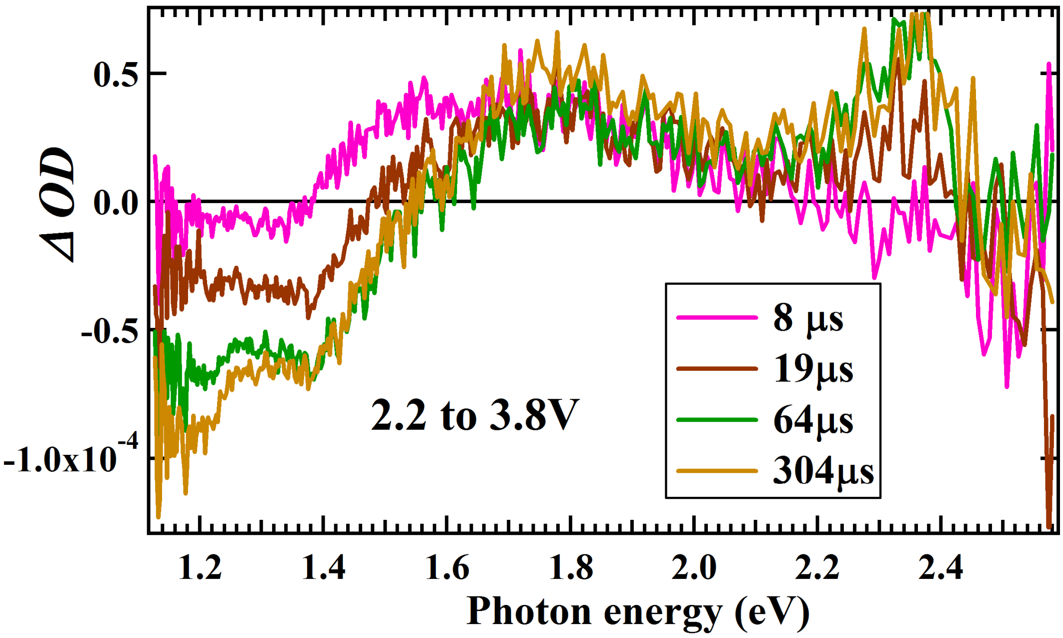

Figure S7 | Time-resolved BM spectra. Time-resolved BM spectra of the SY-LEC for the bias raised from 2.2 V to 3.8 V obtained from the device different from the one shown in the main text.

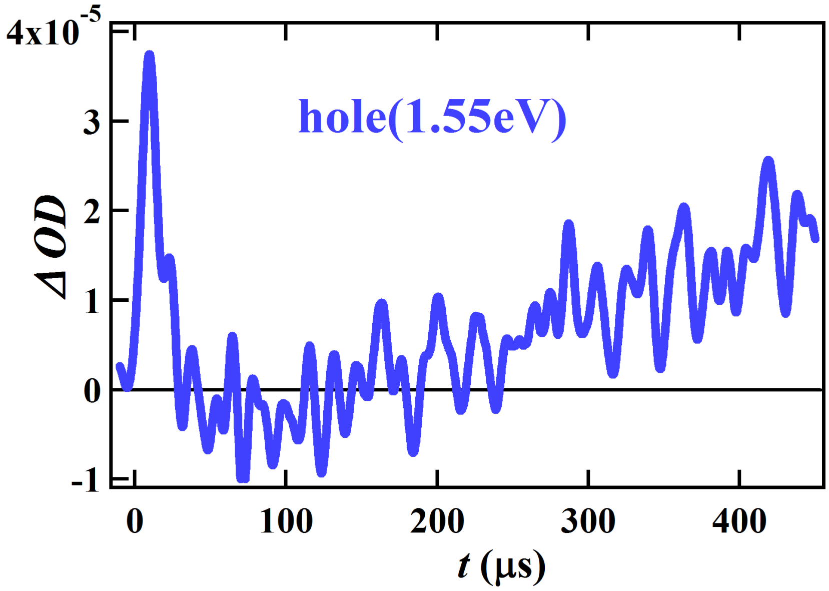

Figure S8 | Enlarged view of the transient response of hole signal. Enlarged view of the transient response of the BM signal measured at 1.55 eV for the hole carriers shown in Fig. 4e.
